# HPLC–MS^2^ Analysis of Chlorophylls in Green Teas Establishes Differences among Varieties

**DOI:** 10.3390/molecules27196171

**Published:** 2022-09-20

**Authors:** Marta Herrera, Isabel Viera, María Roca

**Affiliations:** Food Phytochemistry Department, Instituto de la Grasa, Consejo Superior de Investigaciones Científicas (CSIC), Pablo de Olavide University Campus, Building 46, Carretera de Utrera km. 1, 41013 Sevilla, Spain

**Keywords:** Matcha, Sencha, Gyokuro, green tea, chlorophyll, pheophytin, pheophorbide, oxidized chlorophyll, HPLC, mass spectrometry

## Abstract

Green teas are nonfermented teas, the quality of which is measured by the green color. However, this category encompasses a high number of tea varieties that differ in cultivation and processing. For example, leaf or stem/bubble tea, plants cultivated under a light or shadow regime, powdered or unpowdered tea, etc. These variables determine the different qualities among green teas (Matcha, Sencha, Gyokuro, etc.) and consequently their different values on the market. Our purpose is to determine if these variables can exert an influence on the chlorophyll profile and to establish a characteristic profile for specific green teas. With such an aim, we analyzed the chlorophyll profiles of 6 different green tea varieties via HPLC-*hr* ESI/APCI–MS^2^ and identified up to 17 different chlorophyll compounds. For the first time, 13^2^-hydroxy-chlorophylls, 13^2^-hydroxy-pheophytins, and 15^1^-hydroxy-lactone-pheophytins have been identified in green teas. Shadow teas (Matcha and Sencha) and light-regimen green teas can be statistically differentiated by the total chlorophyll content and the *a*/*b* ratio. However, only Matcha tea contains a higher proportion of chlorophylls *a* and *b* among the green tea varieties analyzed, justifying the higher quality and price of this variety. Other chlorophyll metabolites (pheophytins, pyropheophytins, and oxidized chlorophylls) are indicative of the various processing and storage conditions.

## 1. Introduction

Tea is a very appreciated beverage prepared with the leaves of *Camellia sinensis* L. plant, and it is now the second most-consumed drink in the world, after water. Several processing techniques can be applied to obtain different tea formulations. Thus, the most commonly consumed tea products are divided into white, green, oolong, and black teas, based on the degree of fermentation (nonfermented, partially fermented, and fully fermented). Green tea is nonfermented tea, the fresh leaves of which are subjected to heat treatment to prevent the activation of their oxidative enzymes. Therefore, the green tea market considers the intensity of the green color as one of its quality indexes because it is a measurement of how high the tea’s grade is [1]. There are several varieties of green tea that can be obtained from *Camellia sinensis* L., such as Sencha (which is a nonshadow tea) or Gyokuro (a shadow tea), which are leaf-type teas. However, probably the highest quality of green tea is Matcha due to the special processing of the plant [2]. Matcha leaves are harvested after the long-term shade treatment (at least 20 days) of the tea bushes, and the whole tea leaf is powdered, facilitating consumption. Because of the processing of Matcha, its consumption results in the ingestion of a higher amount of several bioactive compounds normally present in tea, such as catechins, amino acids, polyphenols, theanine, caffeine, and obviously, chlorophylls [3]. Interestingly, most of the named compounds have been shown to exert a positive effect on human health, including anti-inflammatory, antibacterial, cholesterol-lowering, antioxidant capacity, and antimutagenic effects [4]. This has caused an increase in interest in Matcha tea production as a potential food to help in the maintenance of health and the prevention of diseases, and it is now the most expensive green tea in the world [5,6]. 

As chlorophylls are an indicator of green tea quality, different research lines have addressed the study of these compounds in green teas. Different works have dealt with the identification and quantification of chlorophylls in green tea through common spectroscopic methods [7,8], and more recently, new evaluation methods have been developed. In this line, and to improve the quality through the increment in chlorophylls, new estimative technologies have been set up for green tea, such as hyperspectral reflectance and deep learning [9], visible–near-infrared hyperspectral imaging technology [10], and ultra-performance convergence chromatography [11]. Chlorophyll content has been also proposed as an indicator of green tea freshness, and therefore, it has been evaluated with respect to storage conditions and the best packaging options [8]. Even the chlorophyll metabolism in green tea postharvest has been investigated at the enzymatic [12] and the gene-expression level [13] to maximize the quality of its color.

Although there exist some methods for assessing the quality of green teas, they usually do not distinguish between Matcha tea and the rest of green tea varieties [8], which present different health-promoting effects, and which sell at different prices. This lack of agreed guidelines could lead to adulteration, such as selling other green teas as Matcha when they do not fulfil the requirements for this category—for example, steamed green tea powder [14]. ISO-11287:2011 [15] establishes the chemical requirements for green tea. However, with the exceptions that the color should have the characteristics of green tea and that no coloring agent should be added, no standards for chlorophyll content or profile have been declared. Some sensorial methods have also been described to assess Matcha tea quality, but these are based on taste or aroma quality indicators, not on the content of bioactive compounds with health-promoting effects [10]. 

**Figure 1:**
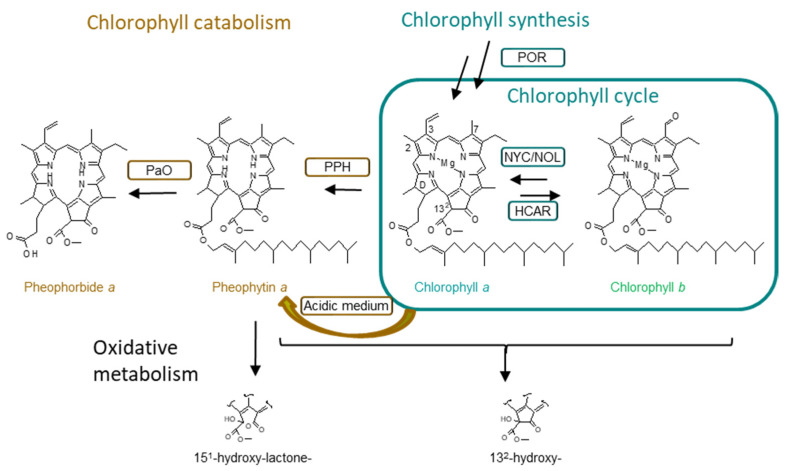
Chlorophyll synthesis (including chlorophyll cycle), catabolism, and oxidative metabolism, adapted from [17]. Formation of pheophytin from chlorophyll in acidic medium. POR stands for protochlorophyllide *a* oxido-reductase, NYC/NOL stands for nonyellow coloring and NYC1-like, PPH stands for pheophytinase, and PaO stands for pheophorbide *a* oxygenase.

As stated before, one of the peculiarities of Matcha tea is that the plants are shaded for a few weeks before harvesting, which makes a difference when compared with other green teas that are grown without using any artificial shade [2]. As photosynthesis is the process that sustains photosynthetic organisms, light is the most important factor influencing plant growth and productivity. For that reason, plants have developed a high capacity to adapt their metabolisms to changes in light intensity [16]. One of these adaptations consists not only in an increase of the total amount of chlorophyll molecules, but also in rearrangements of the photosynthetic apparatus through the interconversion of chlorophyll *a* to chlorophyll *b* through the “chlorophyll cycle” (Figure 1). Thus, the aim of this work is to characterize authentic and high-quality Matcha tea based on the amounts and profiles of its chlorophylls and chlorophyll derivatives, in comparison with other green teas cultivated under shadow and nonshadow conditions. 

## 2. Results

### 2.1. Chlorophyll Profile in Green Tea

A total of 17 different chlorophyll compounds have been identified in green tea through a targeted approach using HPLC coupled to *hr*-MS^2^ spectrometry, and the chromatographic and mass spectrometry characteristics are shown in Table 1. The obtained data regarding the identification of each pigment agree with previous characterizations [18,19], and the analysis was developed using authentic standards. Most of the studies conducted on green tea have been based on chlorophyll *a* and *b* content [9,11,20], which should be the main chlorophyll pigments. In addition, other analytical studies have also identified dephytylated (chlorophyllide and pheophorbide) and phytylated (pheophytin) chlorophyll derivatives [7,13,21]. As shown in Figure 1, these chlorophyll compounds are considered intermediates of the natural metabolism in photosynthetic tissues, specifically during the catabolic pathway [22]. Additionally, pyropheophytin *a* has been also identified, in agreement with previous reports [7]. The decarboxylation from pheophytin to form pyropheophytin is a chemical reaction. However, to the best of our knowledge, this is the first time that oxidized chlorophylls have been identified in green tea leaves. Specifically, we have identified 13^2^-hydroxy-chlorophyll (*a* and *b*), 13^2^-hydroxy-pheophytin (*a* and *b*), and 15^1^-hydroxy-lactone-pheophytin *a* (Table 1). Although their formation could be attributed to peroxidase [23], it cannot be ruled out that the chemical originated during processing. 

Table 2 shows the quantification of each chlorophyll compound identified in the different green tea samples in reference to dry weight. The total chlorophyll content obtained is in line with that found in previous studies although our extraction method resulted in slightly more exhaustive findings for all of the studied samples. Previous studies regarding the chlorophyll content in green teas have obtained amounts in the range of 1000 mg/kg [8,20] and up to 4000 mg/kg [24], while for Matcha samples, the literature describes amounts of up to 15,000 mg/kg [10,24]. In any case, based on the total amount of chlorophylls, two separate groups can be clearly distinguished. Shadow green tea (Matcha and Gyokuro) samples showed a statistically significant (*p* < 0.05) higher number of total chlorophylls compared with the other green tea samples. This is in accordance with the literature since it has been widely described that tea grown under shade conditions contains a higher content of total chlorophylls than tea processed with other techniques [3,14,21,25,26]. Recently, insights have been provided into the regulatory mechanisms responsible for the induced synthesis of chlorophyll by shading in green tea leaves. CsHY5 is a transcription factor that acts downstream to photoreceptors directly affecting the transcription of light-induced genes and specifically biosynthetic genes [27]. In green tea leaves, a decrease in light intensity significantly downregulates the expression of CsHY5, which leads to the enhanced expression of CsPOR (protochlorophyllide oxidoreductase) [20]. POR is the key regulatory enzyme in the chlorophyll synthesis pathway (Figure 1). The activation of this pathway explains the obtained results, in which shadow and nonshadow green teas can be statistically differentiated by their total chlorophyll contents. 

A detailed analysis of the chlorophyll profile in the green tea samples (Table 2) evidenced that, for almost all of the samples analyzed, chlorophyll *a* and *b* quantitatively represented the main chlorophyll compounds. It is important to highlight that, among all of the chlorophylls identified in Table 1, only chlorophylls *a* and *b* (and the tiny amounts of chlorophyllides) exhibit green color. Therefore, if the green color is considered a quality parameter of green tea that determines its trade value, the content of chlorophylls *a* and *b* should be as high as possible. Proportionally, Matcha tea contained between 62 to 81% of chlorophylls *a* and *b*, statistically higher (*p* < 0.05) than the other green teas (55–30%). Interestingly, in Gyokuro, despite being a shadow tea, the proportional contents of green chlorophylls are statistically lower than those in Matcha tea. At some point, it seems that the grinding of Matcha leaves avoids the chlorophylls’ degradation into brownish catabolites (Figure 1). This featured profile is important because chlorophyll and chlorophyllide are involved in some typical tea characteristics, such as the green color infusion and the grassy taste [28], which could improve consumer acceptance. Therefore, the proportional contents of chlorophylls *a* and *b* seem to stand as quality criteria for Matcha tea. Pheophorbides and pheophytins are brownish chlorophylls, and taking into consideration that the teas’ green color is considered a high-quality attribute, their presence leads to a low-quality green color [21]. Both are intermediate catabolites in the chlorophyll degradation pathway (Figure 1) [7], and therefore, their relative presence should be noted as evidence of an active metabolism. In this sense, the relative presence of pheophorbide in powdered green tea (Matcha) was below 0.5%, while in nonpowdered teas (Gyokuro and Sencha), its content increased up to 4.5%. Somehow, it seems that the manufacturing process, which includes a grinding step, avoids the formation of pheophorbides. However, pheophytins, besides being chlorophyll catabolites produced by pheophytinase [22], are also easily formed from chlorophylls under acidic conditions (Figure 1), and its appearance in processed foods has been widely described. During manufacturing, the release of organic acids from cellular organelles produces the conversion of chlorophyll *a* into pheophytin *a* [7]. In the analyzed green tea samples, the relative content of pheophytin (*a* and *b*) varied from 4% in Matcha to 69% in green tea. The random presence of pheophytins in the different types of green tea that we analyzed can only be understood as a reflection of the processing conditions. As an example, pyropheophytins are formed by the decarboxylation of pheophytin under heating conditions, and therefore their appearance is associated with thermal processing [29]. In green teas, pyropheophytins (Table 2) represented between 0.7 to 3%, correlating well with pheophytin content. As it has been proposed previously, pyropheophytins are probably formed during tea production when tea leaves are steamed to inactivate the endogenous enzymes [30]. In this line of thinking, the biochemical origin of the oxidized chlorophyll metabolites (13^2^-hydroxy- chlorophylls and pheophytins and 15^1^-hydroxy-lactone-pheophytins) is still under discussion. In higher plants, different enzymatic complexes have been assumed as being responsible for such oxidation (lipoxygenase and/or peroxidase) [31]. However, most of the studies link the formation of oxidized chlorophylls to a chemical reaction in an oxidative ambient. Therefore, oxidized chlorophylls are commonly associated with processed and stored foods [32,33]. Considering the results obtained in Table 2, the oxidized chlorophylls (13^2^-hydroxy- chlorophylls and pheophytins and 15^1^-hydroxy-lactone-pheophytin *a*) present in the different green tea varieties should be attributed to the conditions suffered during processing or storage.

Consequently, the final proportion of chlorophyll derivatives will depend on both plant metabolism and manufacturing practices. This is important, as Matcha tea’s quality results from the pre-harvesting conditions and the post-harvesting manipulation, which must be adequate in order to avoid the formation of undesired derivatives and to maintain the tea’s quality and price [13]. 

### 2.2. Influence of Shadow Regime in Chlorophyll Green Tea

**Figure 2:**
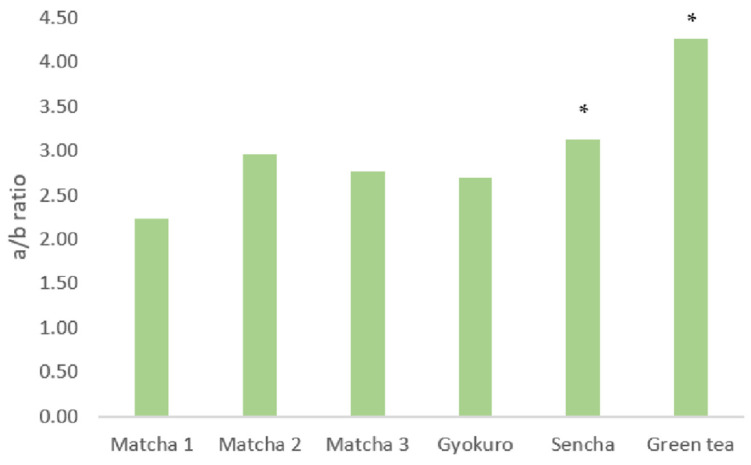
Chlorophyll *a*/*b* ratios among the different studied green teas. (* indicates a statistically significant difference *p* < 0.05).

Figure 2 represents the *a/b* series ratio in all of the analyzed green teas, and Matcha and Gyokuro teas maintain an *a*/*b* ratio of around 2.5, while the rest of the green teas exhibit a ratio of around 3.5. Therefore, this ratio can statistically (*p* < 0.05) distinguish between shadow (Matcha and Gyokuro) and nonshadow green teas. This difference in the *a*/*b* ratio is consistent with previous results [13,14,20]. Indeed, researchers have previously proposed the use of the series *a*/*b* ratio to assess Matcha tea quality and as a possible method of distinguishing potential adulterations of green tea [14]. However, our data point out that such a pattern is nonexclusive to Matcha tea, but it is a characteristic feature among green tea plants cultivated under a shadow regimen. It is known that the light-harvesting complexes (LHC) are rich in chlorophyll *b*, while the centers of reaction are rich in chlorophyll *a* [34]. Therefore, under high irradiance conditions, organisms reduce their LHC to capture as few photons as possible so as to avoid an excess of excitation energy. The consequence is a reduction in the proportion of the chlorophyll *b* series. On the contrary, under shadow conditions, the organisms need to capture as much light as possible, increasing their LHC size, and thus synthesizing proportionally more chlorophyll *b* than *a* [35,36,37]. Consequently, plants grown under high-light conditions will show a normal 3:1 chlorophyll *a* to *b* ratio, while plants grown under low-light conditions will promote the higher production of chlorophyll *b*, showing a lower *a*/*b* proportion [38,39].

## 3. Materials and Methods

All of the procedures were carried out under green light so as to avoid the photooxidation of chlorophylls.

### 3.1. Raw Materials

The study was carried out with 6 different commercial green samples which were declared as including 3 authentic Matcha teas, 1 Gyokuro tea, and 2 green teas (Hojicha and Sencha) cultivated under nonshadow conditions. All of them were bought at specialized tea shops and stored following manufacturer instructions until their analysis. 

### 3.2. Chemicals and Reagents

Ammonium acetate (98%) was supplied by Sigma-Aldrich Chemical Co. (Madrid, Spain). Acetone and methanol (analysis grade) were supplied by VWR BDH Chemicals (Visalia, CA, USA), and purified water was obtained from a Milli-Q water purification system (Millipore, Milford, MA, USA). Chlorophyll standards (chlorophylls *a* and *b*, pheophorbide, and pheophytin *a*) were supplied by Cymit S.L., and the other chlorophylls (chlorophyllide, pyropheophytin *a*, 13^2^-hydroxy-pheophytin *a*, and 15^1^-hydroxy-lactone pheophytin *a*) were obtained following Chen et al. [18,19].

### 3.3. Determination of Humidity

A moisture analyzer, OHAUS MB25 (Nänikon, Switzerland), was used to determine the percentage of moisture in the foods.

### 3.4. Chlorophyll Extraction 

As green tea is a dry powder, the raw materials (0.1 g) were hydrated with 0.1 g of distilled water before extraction. The methodology was based on previously published acetone extraction protocols [40], with some modifications. Briefly, pre-hydrated samples were extracted with 1 mL acetone and homogenized in an ultrasonic bath (5 min, 720 W). Next, samples were filled up to 12 mL acetone, vortexed, and stirred at 4500 rpm for 5 min. The upper acetone phase was collected, and the extraction was repeated (no less than 4 extractions) until no more color could be extracted. The combined acetone extracts were evaporated on a rotatory evaporator, and the dry residue was dissolved in 10 mL of acetone and analyzed by HPLC. Each sample was extracted in triplicate.

### 3.5. Chlorophyll Identification by ESI/APCI-hr-HPLC–MS^2^

Following previous protocols [18,19], the chromatographic separation was performed in a Dionex Ultimate3000RS UHPLC (Thermo Fisher Scientific, Waltham, MA, USA). The elution gradient for separation was followed as described by Chen et al. (2015a) with the following mobile phases: A, water/1 M ammonium acetate in water/methanol (1:1:8, *v*/*v*/*v*) and B, methanol/acetone (1:1, *v*/*v*). The flow rate was 1.0 mL/min, and the injection volume was 50 µL. The HPLC–APCI-hrQqTOF operated for mass analysis using a micrOTOF-QII high-resolution time-of-flight mass spectrometer (HR-ToF) with Qq-ToF geometry (Bruker Daltonics, Bremen, Germany) equipped with an APCI source for nonpolar chlorophylls and an electrospray ionization (ESI) interface for polar chlorophylls. Mass spectra were acquired in bbCID mode, and the multitarget screening was performed using TargetAnalysis 1.2 software (Bruker Daltonics, Bremen, Germany). Bruker Daltonics DataAnalysis 4.1 allowed for the data evaluation, and we set the tolerance limits below 5 ppm for mass error and below 50 for mSigma value. The experimental values of mass-to-charge ratio and isotopic pattern corresponding to those elemental compositions were compared with the theoretical data, obtaining the corresponding mass error and mSigma values. The consistency of the elemental composition and formulas of the experimental product ions were checked by applying the same procedure to the product ions with the assistance of the SmartFormula3D algorithm [18,19].

### 3.6. Chlorophyll Quantification by HPLC–UV–Visible 

Reversed-phase HPLC with a Hewlett-Packard HP 1100 liquid chromatograph was used to separate the pigments. A Mediterranea Sea18 column (200 × 4.6 mm, 3 μm particle size) was used (Teknokroma, Barcelona, Spain), protected by a guard column (10 × 4.6 mm) packed with the same material. The elution gradient for separation was followed as described by Chen et al. (2015a) with the following mobile phases: A, water/1 M ammonium acetate in water/methanol (1:1:8, *v*/*v*/*v*) and B, methanol/acetone (1:1, *v*/*v*). The online UV–visible spectra were recorded from 350 to 800 nm with the photodiode–array detector (Agilent, CA, USA), and sequential detection was performed at 410, 430, 450, and 666 nm. An LC HP ChemStation (Rev.A.05.04; Santa Clara, CA, USA) was used for data collection. Calibration curves (amount vs. integrated peak area) were calculated for the quantification of pigments. The calibration equations were obtained by least-squares linear regression analysis over a concentration range according to the observed levels of these pigments in the samples. LOD and LOQ were determined for each chlorophyll standard. The absence of the matrix effect was also proved. 

### 3.7. Statistical Analysis

Three replicates of each sample were carried out. Data were expressed as means ± standard deviation (SD). Statistical differences were denoted for the individual chlorophyll derivatives, and the *a*/*b* ratio was determined using one-way analysis of variance (ANOVA). Tukey’s multiple-range test was used as a *post hoc* comparison procedure of statistical significance (*p* < 0.05). OriginPro 2020b software was used for the statistical studies.

## 4. Conclusions

Although further studies are required to establish the certainty of our proposal, the data presented in this work serve as a confirmation of the use of the series *a*/*b* ratio as an indication of Matcha quality, and, moreover, the quality of other high-quality tea grown under shade conditions as well. Furthermore, we have found that it is determinant to analyze not only the *a* and *b* chlorophyll series individually, but also the whole profile of chlorophyll derivatives in teas to establish a precise approach for identifying real Matcha tea. 

## Figures and Tables

**Table 1:** Chromatographic and mass spectrometry characteristics of chlorophylls identified in green teas.

Pigment	t_R_ (Min)	Molecular formula	[M + H]^+^ (*m*/*z*) Calcd./Meas.	Mass Error (ppm)	mSigma	Main Product ions (*m*/*z*)
Chlorophyllide *b*	2.2	C_35_H_32_MgN_4_O_6_	625.2245/629.2248	−0.4	26.5	569.2046
Chlorophyllide *a*	4.9	C_35_H_34_MgN_4_O_5_	615.2452/615.2424	4.6	23.8	500.2147
Pheophorbide *b*	7.0	C_35_H_34_N_4_O_6_	607.2551/607.2532	3.2	21.6	547.2332
Pheophorbide *a*	8.7	C_35_H_36_N_4_O_5_	593.2758/593.2734	4.1	27.4	533.2523
13^2^-hydroxy-chlorophyll *b*	14.5	C_55_H_70_MgN_4_O_7_	923.5168/923.5213	−4.9	40.1	569.1837
Chlorophyll *b*	17.0	C_55_H_70_MgN_4_O_6_	907.5219/907.5202	1.8	39.7	569.2073
Chlorophyll b´	17.5	C_55_H_70_MgN_4_O_6_	907.5219/907.5217	0.5	28.8	569.2025
13^2^-hydroxy-chlorophyll *a*	18.5	C_55_H_72_MgN_4_O_6_	909.5375/909.5377	−0.2	38.1	555.2257
13^2^-hydroxy-pheophytin *b*	19.2	C_55_H_72_N_4_O_7_	901.5474/901.5436	−4.21	25.7	607.2535
Chlorophyll *a*	19.8	C_55_H_72_MgN_4_O_5_	893.5426/893.5468	−4.7	34.5	555.2234
Chlorophyll a´	20.1	C_55_H_72_MgN_4_O_5_	893.5426/893.5421	0.6	25.4	555.2249
15^1^-hydroxy-lactone-pheophytin *a*	22.1	C_55_H_74_N_4_O_7_	903.5630/903.5624	0.7	42.9	549.2510
Pheophytin *b*	26.2	C_53_H_72_N_4_O_6_	885.5525/885.5515	1.1	12.8	547.2305
13^2^-hydroxy-pheophytin *a*	26.1	C_55_H_74_N_4_O_6_	887.5681/887.56420	4.4	49.2	593.2723
Pheophytin *a*	28.6	C_55_H_74_N_4_O_5_	871.5732/871.5723	1.0	13.0	593.2703
Pheophytin a´	29.1	C_55_H_74_N_4_O_5_	871.5732/871.5702	−2.3	45.4	593.2711
Pyropheophytin *a*	30.7	C_53_H_72_N_4_O_3_	813.5677/813.5696	−2.3	9.3	535.2647

**Table 2:** Chlorophyll profile in green teas (mg/Kg dw ± SD).

	Matcha 1	Matcha 2	Matcha 3	Gyokuro	Sencha	Green Tea
TOTAL	12,111.19 ± 323.42	13,257.10 ± 728.80	17,262.65 ± 2154.56	119,74.62 ± 2662.68	5608.84 ± 352.37 *	1060.22 ± 2.08 *
Chlorophyllide *b*	31.91 ± 3.43					0.66 ± 0.04
Chlorophyllide *a*	26.57 ± 5.31	25.01 ± 4.03	23.00±3.27	38.53 ± 1.74	61.47 ± 13.79	0.45 ± 0.02
Pheophorbide *b*		14.77 ± 2.88		7.04 ± 1.21		
Pheophorbide *a*	23.07±0.06	56.93 ± 14.25	54.45 ± 5.64	159.33 ± 34.26	243.68 ± 59.71	6.15 ± 0.69
Chlorophyll *b*	3618.31 ± 138.55	3212.32 ± 165.36	4395.69 ± 449.67	2917.18 ± 596.12	1278.77 ± 71.37	197.79 ± 0.08
Chlorophyll *a*	6192.68 ± 127.09	5807.11 ± 502.59	6220.71 ± 682.86	3814.98 ± 834.68	1840.28 ± 66.88	122.35 ± 0.31
Pheophytin *a*	2114.12 ± 56.56	4005.67 ± 67.14	6391.76 ± 941.74	4705.64 ± 1112.35	2105.20 ± 269.70	728.30 ± 5.58
Pheophytin *b*	51.76 ± 9.83	135.38 ± 2.93	176.21 ± 72.55	317.52 ± 105.11	78.61 ± 16.74	2.19 ± 0.04
15^1^-hydroxy-lactone-pheophytin *a*		84.40 ± 4.13	106.69 ± 14.21	12.99 ± 5.95	1.50 ± 0.25	11.85 ± 0.01
13^2^-hydroxy-chlorophylls ^a^	2330.86 ± 111.99	448.01 ± 20.68	1106.67 ± 343.86	144.12 ± 71.20	59.41 ± 21.81	94.29 ± 1.05
Pyropheophytin	94.96 ± 7.46	120.10 ± 4.39	430.24 ± 53.08	212.16 ± 54.10	101.99 ± 30.71	35.94 ± 0.12

* indicates a statistically significant difference *p* < 0.05. ^a^ 13^2^-hydroxy-chlorophylls stands for 13^2^-hydroxy-chlorophylls and 13^2^-hydroxy-pheophytins *a* and *b*. Empty cells indicate that the compound has not been detected.

## Data Availability

Not applicable.

## Sample Availability

Samples of the compounds are not available or not from the authors.
